# The Deficiency of Indoleamine 2,3-Dioxygenase Aggravates the CCl_4_-Induced Liver Fibrosis in Mice

**DOI:** 10.1371/journal.pone.0162183

**Published:** 2016-09-06

**Authors:** Hideyuki Ogiso, Hiroyasu Ito, Tatsuya Ando, Yuko Arioka, Ayumu Kanbe, Kazuki Ando, Tetsuya Ishikawa, Kuniaki Saito, Akira Hara, Hisataka Moriwaki, Masahito Shimizu, Mitsuru Seishima

**Affiliations:** 1 Department of Informative Clinical Medicine, Gifu University Graduate School of Medicine, Gifu, Japan; 2 First Department of Internal Medicine, Gifu University Graduate School of Medicine, Gifu, Japan; 3 Department of Medical Technology, Nagoya University School of Health Sciences, Nagoya, Japan; 4 Human Health Sciences, Kyoto University Graduate School of Medicine and Faculty of Medicine, Kyoto, Japan; 5 Department of Tumor Pathology, Gifu University Graduate School of Medicine, Gifu, Japan; Centre National de la Recherche Scientifique, FRANCE

## Abstract

In the present study, we examined the role of indoleamine 2,3-dioxygenase (IDO) in the development of CCl_4_-induced hepatic fibrosis. The liver fibrosis induced by repetitive administration with CCl_4_ was aggravated in IDO-KO mice compared to WT mice. In IDO-KO mice treated with CCl_4_, the number of several inflammatory cells and the expression of pro-inflammatory cytokines increased in the liver. In the results, activated hepatic stellate cells (HSCs) and fibrogenic factors on HSCs increased after repetitive CCl_4_ administration in IDO-KO mice compared to WT mice. Moreover, the treatment with l-tryptophan aggravated the CCl_4_-induced hepatic fibrosis in WT mice. Our findings demonstrated that the IDO deficiency enhanced the inflammation in the liver and aggravated liver fibrosis in repetitive CCl_4_-treated mice.

## Introduction

Hepatic fibrosis is a wound-healing response to various chronic hepatic injuries resulting from viral infection (particularly hepatitis B and C), alcohol abuse, drugs, metabolic diseases, or autoimmune diseases [1]. Continued progression of hepatic fibrosis results in liver cirrhosis and may cause chronic hepatic failure or liver cancer [2]. Hepatic fibrosis is characterized by the deposition of extracellular matrix (ECM), including type I and III collagens, glycoproteins and proteoglycans [3]. ECM deposition is increased due to excessive ECM production and reduced ECM degradation in the liver. The most important event in hepatic fibrosis is the activation of hepatic stellate cells (HSCs). Following liver injury of any etiology, HSCs undergo a response activation, which involves the transition of quiescent cells into proliferative and fibrogenic myofibroblasts. Such activated HSCs are then able to produce ECM in the liver [1].

Indoleamine 2,3-dioxygenase (IDO) has been identified as a powerful immunomodulatory molecule with significant enzymatic activity for catabolism of the essential amino acid l-tryptophan [4,5]. IDO is expressed in epithelial cells, macrophages, and dendritic cells (DCs), and is up-regulated by pro-inflammatory cytokines, including interferon-γ, tumor necrosis factor (TNF)-α and interleukin (IL)-6 [6]. Previous reports indicated that IDO expression of the liver was enhanced in acute hepatitis model [7,8]. IDO activity has been found to greatly impact immune tolerance and immune regulation [9]. The immunosuppressive activity of IDO is derived from inhibition of T cell activation and proliferation in microenvironment, where tryptophan is decreased and tryptophan metabolites are increased [10]. Also, IDO can enhance regulatory T cells (Tregs) activity [11] and suppress host immune response via Tregs [12].

Pro-inflammatory cytokines, such as TNF-α and IL-6, have been shown to increase in the liver of carbon tetrachloride (CCl_4_)-induced hepatitis [13]. Pro-inflammatory cytokines and chemokines stimulate HSCs, which produce ECM in the liver. Thus, liver fibrosis is closely related to liver inflammation. Therefore, IDO induced by various pro-inflammatory cytokines may be involved in the attenuation of liver fibrosis. We indicated in a previous report that IDO attenuated liver injury in an α-galactosylceramide-induced hepatitis model [4]. Moreover, a recent report demonstrated that the inhibition of IDO activity by 1-methyl-_DL_-tryptophan (1-MT) exacerbated CCl_4_-induced liver injury [14]. However, the role of IDO in the development of liver fibrosis remains unclear. In the present study, we examined the effect of IDO on CCl_4_-induced liver fibrosis in mice and demonstrated that the deficiency of IDO aggravates the development of liver fibrosis.

## Materials and Methods

### Mice

Male C57BL/6J wild-type (WT) mice (age, 6–8 weeks) were obtained from Japan SLC (Shizuoka, Japan). IDO-knockout (IDO-KO) mice with a C57BL/6J background were obtained from The Jackson Laboratory (Bar Harbor, ME). Mice were maintained at the specific pathogen free unit under 12 h light-dark cycle at 23°C. Mice were provided free access to food and water. The food was obtained from Japan SLC (Shizuoka, Japan). The study protocols were approved by the Ethics Committee for Animal Experiments of Gifu University. All protocols were in accordance with guidelines established by the National Institutes of Health Guide for Care and Use of Laboratory Animals.

### Animal Experiments

Mice received intraperitoneal injections of a 10% CCl_4_ (Wako, Osaka, Japan) solution in olive oil (1 μg/g body weight) twice a week for 6 weeks, while control mice were administrated only olive oil. In some experiments, mice were intraperitoneally administered with l-tryptophan (Wako, Osaka, Japan) or l-kynurenine (Sigma-Aldrich, St Louis, MO) twice a week for 6 weeks. Mice were sacrificed by cervical dislocation 7 days after the final administration of CCl_4_, and necropsy was performed. In another group, mice were administered a single dosage of 10% CCl_4_ solution in olive oil (1 μg/g body weight) intraperitoneally and were sacrificed in a similar manner at day 0, day 1, day 3 and day 6 post-administration.

### Histology and Immunohistochemistry

Tissue samples were fixed in 10% buffered formalin and embedded in paraffin. The 4 μm thick sections of the livers were stained with hematoxylin-eosin. To assess collagen deposition (fibrosis), 4 μm thick sections of the livers were processed by Azan staining using a standard histological procedure [15]. Also, 4 μm thick sections of the livers were stained by Picrosirius Red Stain Kit (Polysciences, Philadelphia, PA). The degree of fibrosis was evaluated semi-quantitatively using the ImageJ program (National Institute of Health). Immuno-histochemical staining for α-smooth muscle actin (α-SMA) was used to evaluate activation of HSCs in the liver, as previously described [16,17]. Briefly, sections were deparaffinized and treated with 3% hydrogen peroxide to inactivate endogenous peroxidases. Sections were heated in 0.1 M citrate buffer (pH 6.0), using the Pascal Heat Induced Antigen Retrieval System (Dako, Grostrup, Denmark). Non-specific antibody binding sites were blocked in phosphate-buffered saline (PBS, pH 7.4) containing 2% bovine serum albumin (BSA, Wako Pure Chemical Industries, Osaka, Japan) for 30 min. The sections were then incubated with rabbit anti α-actin monoclonal antibody (ab5694, Abcam, Tokyo, Japan) diluted 1/850 in PBS and incubated overnight at 4°C. Sections were incubated with rabbit immunoglobulin antibody (E0432, Dako, Grostrup, Denmark) diluted 1/300 in PBS. α-SMA protein was observed by using a labelled streptavidin-biotin kit (Dako, Grostrup, Denmark) containing biotinylated antibody and peroxidase-labeled streptavidin. The peroxidase binding sites were detected by staining with 3,3”-diaminobenzidine. Finally, samples were counterstained using Mayer’s hematoxylin.

### Sircol^™^ Collagen Assay

The collagen content in the liver tissues was determined using the Sircol ^™^ Collagen Assay (Biocolor Ltd., UK), according to the manufacturer’s protocol. This assay uses Sirius Red, an anionic dye with sulfonic acid side chain groups, which reacts with the basic amino acids present in collagen. Briefly, liver tissues were homogenized and collagen was solubilized in 0.5 M acetic acid. Tissue extracts were incubated with the Sirius Red dye and absorbance was determined with a microplate reader (BIORAD, Hercules, CA) at a wavelength of 540 nm. The amount of collagen was expressed as μg/g wet tissue.

### Analysis of liver transaminase

Hepatocyte damage was assessed at the indicated time points after CCl_4_ injection through the measurement of plasma alanine aminotransferase (ALT) activities using an automated clinical analyzer (BM2250; JEOL, Tokyo, Japan).

### Measurements of l-tryptophan

Liver tissue from the mice was mixed with 6 volume of 10% perchloric acid and homogenized. Next, homogenized liver tissue was subjected sonication by homogenizer (Sonifer: BRANSON, Danbury, CT). After centrifugation, the concentrations of l-tryptophan in the supernatants were measured using HPLC with Brava C18-ODS Column (150 × 4.6mm 3μm; GRACE, Columbia, United States) and a spectrophotometric detector or a fluorescence spectrometric detector as described previously [18]. UV signals were monitored at 280 nm for l-tryptophan. The mobile phase consisted of 2.5% acetonitrile in 0.1 M sodium acetate (pH 3.9) and was filtered through a 0.45-μm-pore HA-type filter obtained from Millipore (Bedford, MA). The flow rate was maintained 0.75ml/min throughout the chromatographic run.

### Hepatic mononuclear cell preparation and flow cytometric analysis

Hepatic mononuclear cells (MNCs) were isolated as previously described [19]. Briefly, the excised liver was cut into small pieces with scissors, pressed through a 200-gauge stainless mesh, and suspended in PBS. Hepatic MNCs were separated from parenchymal hepatocytes and hepatocyte nuclei by Ficoll-Conray (IBL, Gunma, Japan) and washed twice in ice-cold medium. Cell viability and cell numbers were assessed by trypan blue exclusion. For flow cytometry, liver MNCs were stained using a standard protocol. The following Abs were used: FITC conjugated Anti-mouse F4/80 mAb (Clone: BM8) and PE-Cy7 conjugated Anti-mouse CD11b mAb (Clone: M1/70). Samples were acquired on BD FACSCanto2 flow cytometer (BD Biosciences, San Diego, CA).

### MACS cell preparation

CD11b+, CD11b-, CD11c+ and CD11c- cells were isolated by MACS Magnetic Bead columns (Miltenyi Biotec, Bergisch Gladbach, Germany) with antibodies against CD11b and CD11c, according to the manufacture’s instructions. Briefly, the cell pellet was suspended in 0.5ml of PBS, 0.5% bovine serum albumin, 2mM EDTA, followed by addition of anti CD11b or CD11c magnetic beads and incubated at 4°C for 15min. CD11b+, CD11b-, CD11c-/CD11c+, and CD11b-/CD11c- cells were isolated by MACS MagneticBead Column.

### HSCs isolation

HSCs were isolated from the mouse liver as described previously with a minor modification [20]. Briefly, the mouse liver was perfused with Liver Perfusion Medium at 37°C, followed by Liver Digest Medium (Invitrogen, NY). The digested liver was excised and minced with scissors. The resulting suspension was filtered through a stainless steel mesh (150 μm diameter). The suspension was centrifuged at 50 × *g* for 2 min at 4°C. The supernatant, including HSCs, was suspended in a 15% iodixanol (Optiprep, Oslo, Norway) solution. Few percent of 10% iodixanol solution and PBS were then layered on to the cell suspension, and HSCs were corrected at the interface between 10% iodixanol and PBS were corrected after centrifugation at 1,400 × *g* for 20 min at 4°C.

### Quantitative real-time reverse transcription-polymerase chain reaction analysis

Total RNA was extracted from mouse liver tissues or isolated HSCs using Isogen II (Nippongene, Tokyo, Japan) and then transcribed to cDNA using the High Capacity cDNA Transcription Kit (Applied Biosystems, Foster City, CA) according to the manufacturer’s protocol. From each sample, 1 μg of total RNA was used as a template for cDNA synthesis. The resulting cDNA was used as a template for real-time polymerase chain reaction (PCR) conducted using pre-designed primer/probe sets for IDO1, IL1-β, IL-6, TNF-α, CCL2, PDGF-β, Col1a2, ACTA2, Timp-1, and 18S rRNA (Applied Biosystems) and Twinbird Probe qPCR Mix (TOYOBO, Osaka, Japan) for Taqman Gene Expression Assays. 18S rRNA was used as an internal control. Real-time PCR was performed using the Light-Cycler Rapid Thermal Cycler System (Roche Diagnostic Systems, Indianapolis, IN). For evaluation of mRNA expression of CYP1a2, CYP2e1, we analyzed on Light Cycler Rapid Thermal Cycler System using the KAPA SYBR Fast qPCR kit (KAPA BIOSYSTEMS). Primers for RT-qPCR used in this study were as follows;

Cyp1a2-fw: TGGAGCTGGCTTTGACACAG, CYP1a2-rv: CGTTAGGCCATGTCACAAGTAGC, Cyp2e1-fw: AAGCGCTTCGGGCCAG, Cyp2e1-rv: TAGCCATGCAGGACCACGA.

### Statistical analysis

The data are expressed as the mean ± standard error of the mean (SEM). Statistical significance of the differences between two groups were determined using Student’s t-test, and those among three groups were tested using one-way analysis of variance (ANOVA). The criterion for statistical significance was P < 0.05.

## Results

### IDO1 expression and IDO activity were up-regulated after CCl_4_ administration

**Fig 1:**
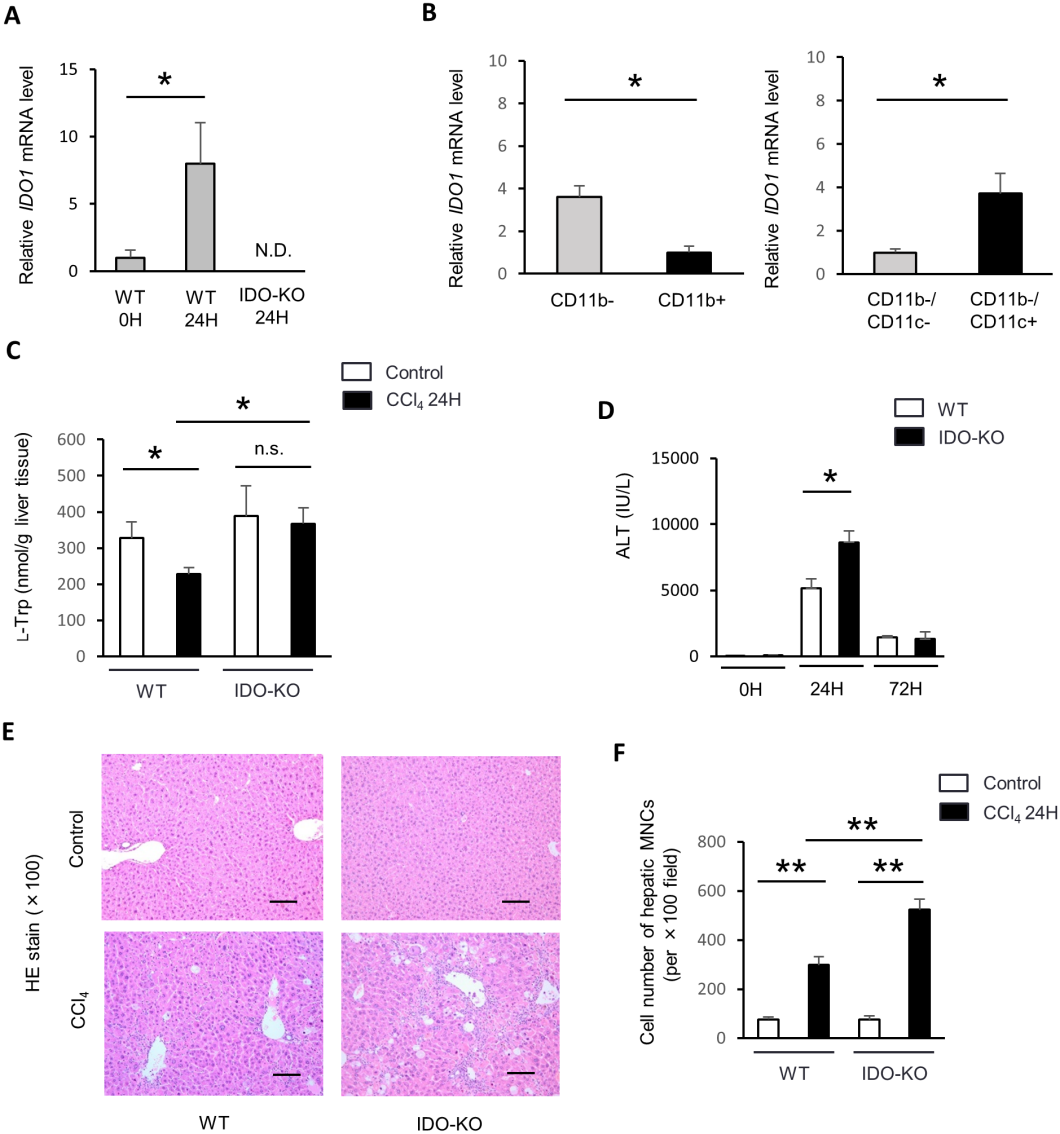
IDO expression and activity were up-regulated after CCl_4_ administration in WT mice. Male C57BL/6J WT mice (n = 3) and IDO-KO mice (n = 3) were treated with CCl_4_ (1 mL/kg, 10% diluted in olive oil). The control group mice (WT mice, n = 3 and IDO-KO mice, n = 3) were injected with olive oil alone. All mice were sacrificed at 24 hours after the administration of CCl_4_ or olive oil alone. (A) mRNA expressions of IDO1 in the liver were measured by quantitative real-time RT-PCR. The results were normalized to the expression of 18S rRNA. (B) mRNA expression of IDO1 in CD11b- cells, CD11b+ cells, CD11c- cells and CD11c+ cells in the liver of CCl_4_ induced injury. WT mice (n = 3) mice were treated with a single CCl_4_ dosage (1 mL/kg, 10% diluted in olive oil). Hepatic MNCs were subjected MACS cell preparation with CD11b- and CD11b+ cells 24 hours after CCl_4_ administration. Also too, hepatic MNCs were subjected MACS cell preparation with CD11c- and CD11c+ cells. (C) The concentrations of l-tryptophan in supernatants of liver homogenates were measured using HPLC (n = 3–5: each group). (D) Serum ALT levels in WT or IDO-KO mice were measured 0, 24, and 72 hours after CCl_4_ administration (n = 3–7: each group). (E) Representative photomicrographs of liver sections at 24 hours after CCl_4_ administration stained with hematoxylin-eosin, ×100 original magnification, Scale bars: 100 μm. (F) The cell number of hepatic MNCs at 24 hours after CCl_4_ administration per ×400 field (n = 3: each group). Each column and error bar represents the mean and SEM, respectively, of results for triplicate samples. * indicate statistically significant differences, at P<0.05.

In a previous study, IDO1 expression was enhanced in the liver of hepatitis model [7]. We measured the expression of IDO1 mRNA in the liver 24 hours after CCl_4_ administration by quantitative real-time RT-PCR. IDO1 mRNA expression was significantly increased in WT mice after CCl_4_ administration (Fig 1A). Meanwhile, IDO1 mRNA expression was not detected in IDO-KO mice. Previously report indicated myeloid CD11c+ dendritic cells express IDO at the inflammatory border [21]. To address which type of cell expresses IDO1 after CCl_4_ administration, we isolated CD11b+ (5.3 ± 1.4 × 10^5^ / liver), CD11b- (25.7 ± 4.9 × 10^5^ / liver), CD11b-/CD11c- (25.5± 4.8 × 10^5^ / liver), and CD11b-/CD11c+ (0.2 ± 0.1 × 10^5^ / liver) cells from hepatic MNCs of CCl_4_-administrated mice using MACS system. Subsequently, we examined the mRNA expression of IDO1 in each cell population using real-time RT-PCR. IDO1 expression was significantly higher in CD11b- cells and CD11b-/CD11c+ cells after the treatment of CCl_4_ (Fig 1B). Therefore, these results indicated that CD11b-/CD11c+ cells mainly expressed IDO1 mRNA after CCl_4_ administration.

IDO is the enzyme which catabolizes l-tryptophan to l-kynurenine. To evaluate the IDO activity, we measured l-tryptophan levels in the liver. l-tryptophan levels in the liver tissue from WT mice were significantly reduced after CCl_4_ administration (Fig 1C). In contrast, l-tryptophan levels in the liver of IDO-KO mice were not reduced after CCl_4_ administration.

### The CCl_4_-induced liver injury and hepatic fibrosis in IDO-KO mice was exacerbated compared to that in WT mice

To evaluate the role of IDO in CCl_4_-induced liver injury, we measured serum ALT activity in WT and IDO-KO mice after single administration of CCl_4_ to WT and IDO-KO. Serum ALT levels in IDO-KO mice significantly increased 24 hours after CCl_4_ injection compared with those in WT mice (Fig 1D). Histological examination also indicated that inflammatory response in IDO-KO was exacerbated compared with that in WT mice after the administration of CCl_4_ (Fig 1E). Cell number of hepatic MNCs was significantly increased in IDO-KO mice compared to WT mice 24 hours after CCl_4_ administration (Fig 1F).

CCl_4_ is metabolized by cytochrome P450 in endoplasmic reticulum of the liver. To dismiss the possibility of the difference between WT mice and IDO-KO in the liver cytochrome level, we investigated the cytochrome level (CYP1A2 and CYP2E1) in the liver of WT and IDO-KO mice. There was no difference of these cytochrome expression levels in the liver between WT mice and IDO-KO mice (S1 Fig).

**Fig 2:**
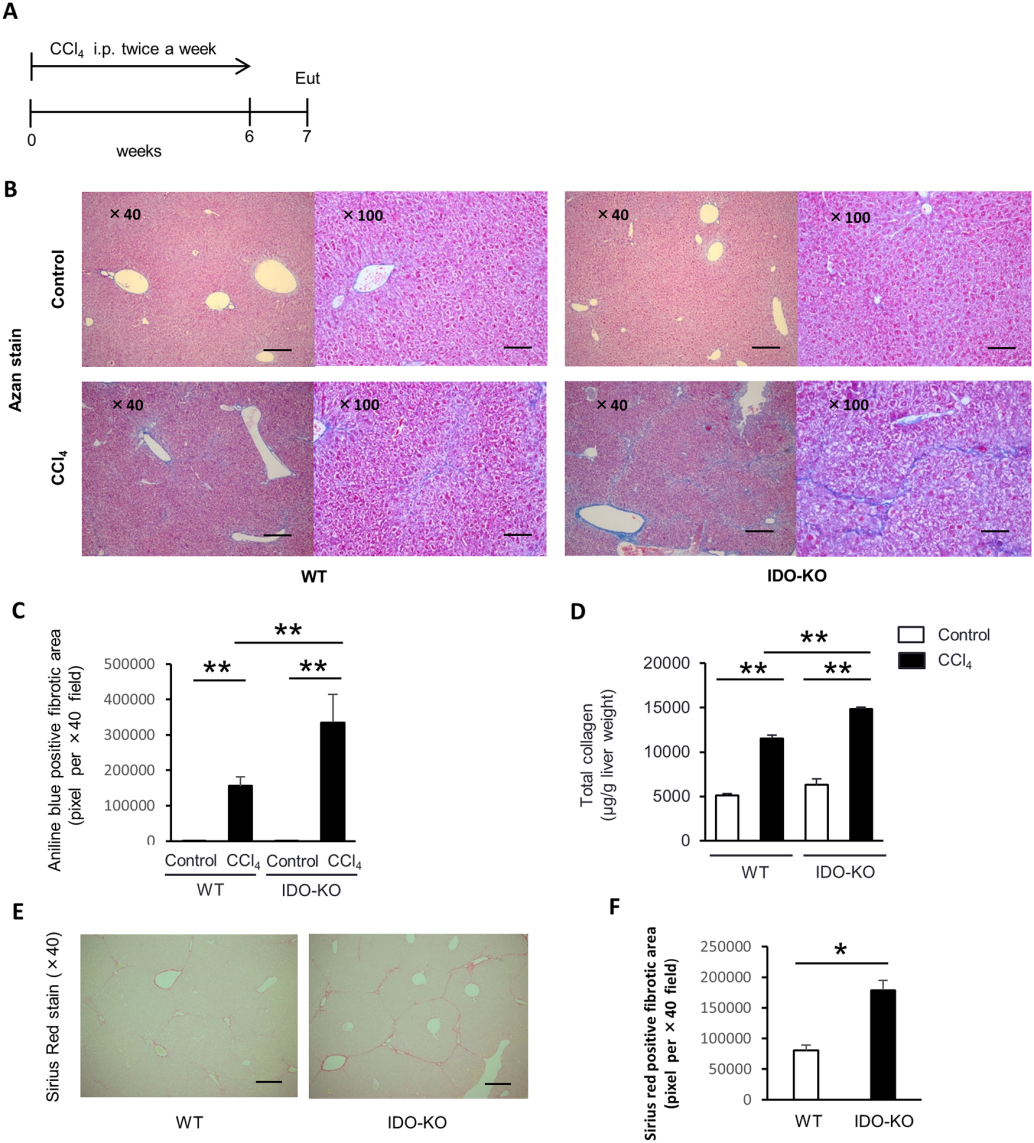
CCl_4_-induced hepatic fibrosis was exacerbated in IDO-KO mice compared to WT mice. Male C57BL/6J WT mice (n = 4) and IDO-KO mice (n = 5) were treated with CCl_4_ (1 mL/kg, 10% diluted in olive oil) twice a week for 6 weeks. All mice were sacrificed at 7 days after the last administration of CCl_4_. The control group mice (WT mice, n = 5 and IDO-KO mice, n = 5) were injected with olive oil alone. (A) The diagram showing experimental design. Eut, euthanasia. (B) Representative photomicrographs of experimental mice liver sections of Azan staining, left panel: ×40 original magnification, Scale bars: 250 μm, right panel: ×100 original magnification, Scale bars: 100 μm. (C) Evaluation of fibrosis was quantified based on the aniline blue-positive fibrotic area in five random fields on the liver tissue sections for each group using ImageJ software. (D) The total collagen content in the liver tissue after the repeatedly CCl_4_ administration was measured using a Sircol^™^ Collagen Assay. (E) Representative photomicrographs of repeatedly CCl_4_-treated mice liver sections stained with Sirius red, ×40 original magnification, Scale bars: 250 μm. (F) Evaluation of fibrosis was quantified based on the Sirius red-positive fibrotic area in five random fields on the liver tissue sections for each group using ImageJ software. Each column and error bar represents the mean and SEM, respectively, of results for triplicate samples. * indicate statistically significant differences, at P<0.05. ** indicate statistically significant differences, at P<0.01.

To evaluate the effect of IDO on CCl_4_-induced hepatic fibrosis, WT mice and IDO-KO mice were treated with CCl_4_ twice a week for 6 weeks. All mice were sacrificed at 7 days after the last administration of CCl_4_. The liver sections were stained by Azan staining. Histological analysis showed that collagen deposition around Glisson’s sheath in the IDO-KO mice increased after CCl_4_ treatment compared to that in WT mice (Fig 2B). Evaluation of fibrosis was quantified based on the aniline blue-positive fibrotic area in five random fields on Azan staining sections of liver from each group. The aniline blue-positive areas in IDO-KO mouse tissue was significantly increased compared to that of the WT mice (Fig 2C). Because hepatic fibrosis is the result of the accumulation of ECM including collagen, we then measured the level of total collagen in the liver tissue of WT mice and IDO-KO mice treated with CCl_4_. The total collagen level in the livers from the IDO-KO mice was significantly increased compared to those from WT mice after CCl_4_ treatment (Fig 2D). Aniline blue stains not only collagen-deposition area but also basement membrane mucin in Azan staining method. Sirius red staining shows only collagenous structures as brilliant red-positive area. Therefore, we also stained the liver sections by Sirius red. The Sirius red-positive areas in IDO-KO mouse tissue was significantly increased compared to that of the WT mice (Fig 2E and 2F). Moreover, 1-MT treatment also promoted the liver fibrosis induced by repetitive administration with CCl_4_ (S2 Fig).

### mRNA expression of pro-inflammatory cytokines and fibrogenic factors after single administration of CCl_4_

**Fig 3:**
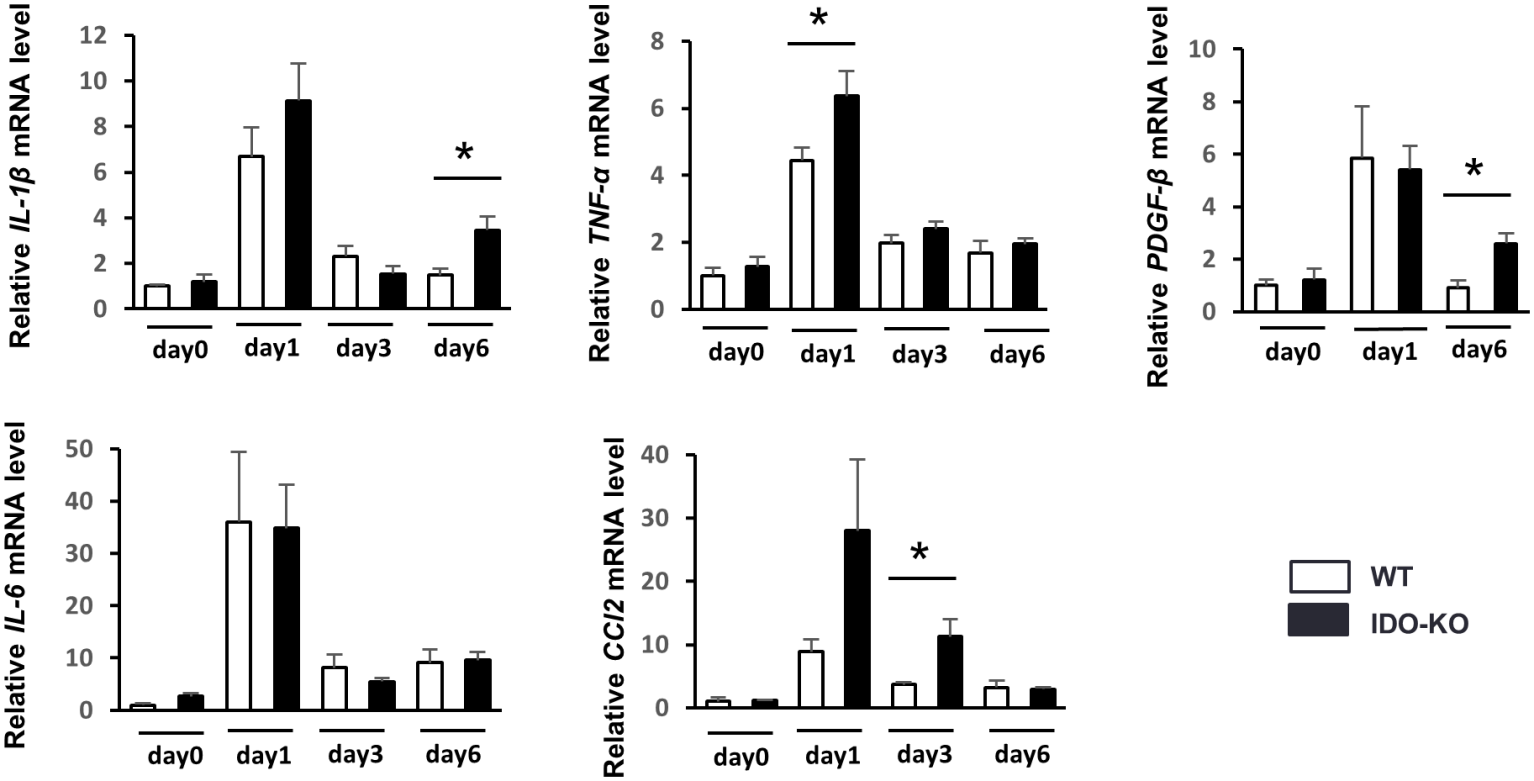
mRNA expression of pro-inflammatory cytokines and fibrogenic factors after single administration of CCl_4_. WT mice and IDO-KO mice were treated with a single CCl_4_ dosage (1 mL/kg, 10% diluted in olive oil) (n = 5: each group). The control group (WT mice and IDO-KO mice) was injected with olive oil alone (n = 3: each group). The relative expression levels of IL-1β, TNF-α, IL-6, CCL2, and PDGF-β mRNA in the liver were measured on day 0, 1, 3 and 6 after CCl_4_ administration using quantitative real time RT-PCR. The results were normalized to the expression of 18S rRNA. Each column and error bar represents the mean and SEM, respectively, of results for triplicate samples. * indicate statistically significant differences, at P<0.05.

As previously reported, HSCs are activated by paracrine stimulation with various cytokines and chemokines released from hepatic parenchymal cells [22], Kupffer cells [23], neutrophils, and platelets [24]. Therefore, we conducted a detailed analysis of the mRNA expression of intrahepatic pro-inflammatory cytokines (IL-1β, TNF-α, IL-6) 0, 1, 3, and 6 days after CCl_4_ single injection using real time RT-PCR. Among pro-inflammatory cytokines, the mRNA expression of TNF-α in hepatic tissue was significantly higher in IDO-KO mice than in WT mice 1 day after administration of a single CCl_4_ dosage (Fig 3). The mRNA expression of IL-1β was also increased in IDO-KO mice compared to WT mice 6 days after CCl_4_ injection. Monocyte and macrophage migrate toward inflamed tissues under the influence of CCL2 and produce TNF-α in inflamed tissues [25]. Therefore, we analyzed the mRNA expression of CCL2. The mRNA expression of CCL2 in hepatic tissue was significantly higher in IDO-KO mice than in WT mice. Moreover, we measured mRNA expression of fibrotic factor (PDGF-β) in hepatic tissue. mRNA expression of PDGF-β was significant higher in IDO-KO mice than WT mice 6 days after administration of single CCl_4_ dosage (Fig 3).

### F4/80+CD11b+ cells was significantly increased in IDO-KO mice compared to WT mice after CCl_4_ administration

**Fig 4:**
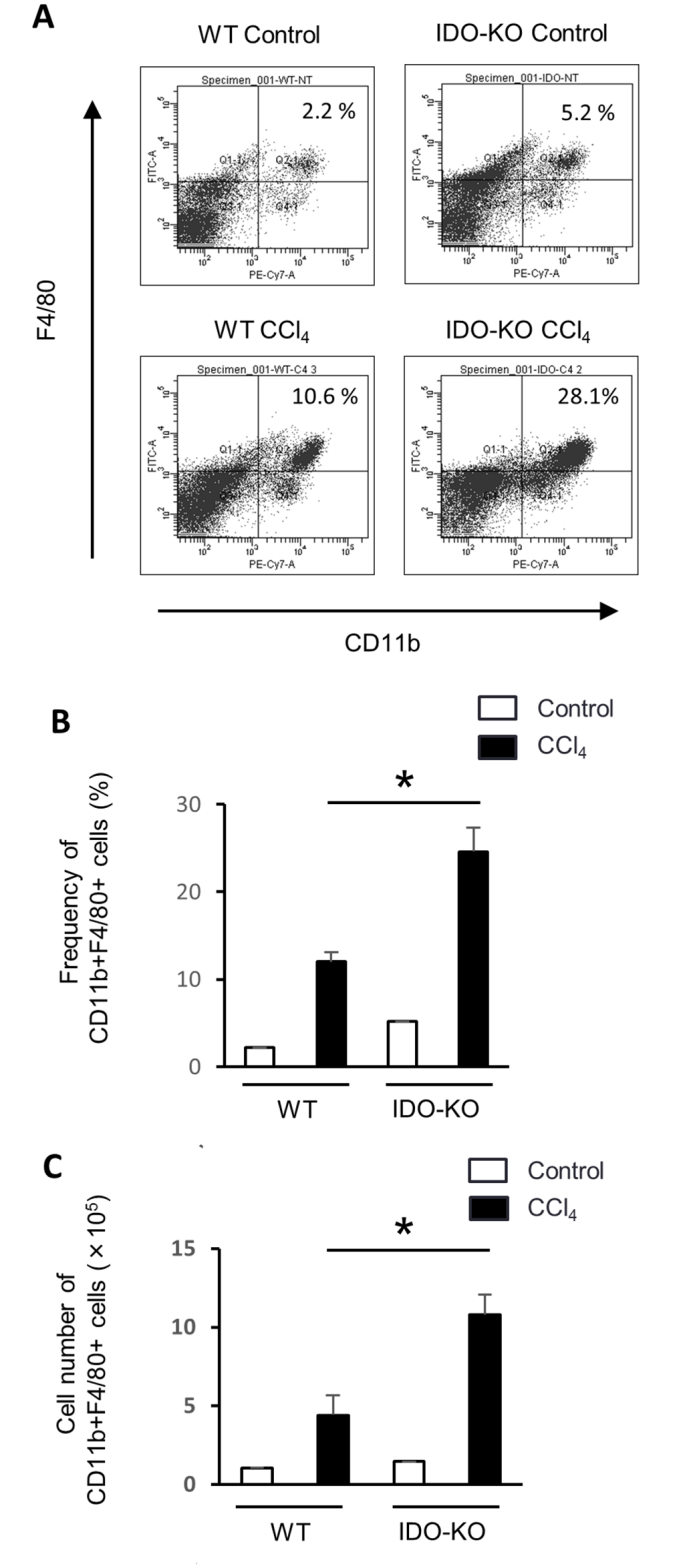
The frequency and cell number of F4/80+CD11b+ cells after single administration of CCl_4_. (A) FACS analysis of F4/80+CD11b+ cells in hepatic MNCs after the administration with CCl_4_. WT mice (n = 3) and IDO-KO mice (n = 3) were treated with CCl_4_ injection (1 mL/kg, 10% diluted in olive oil). Control group (WT mice and IDO-KO mice) were administrated with olive oil alone. All mice were sacrificed at 24 hours after the administration of CCl_4_ or olive oil alone. Representative flow cytometry data were presented. (B) The frequency of F4/80+CD11b+ cells in the liver of WT and IDO-KO mice treated with CCl_4_ administration. (C) The total cell number of F4/80+CD11b+ cells in the liver of WT and IDO-KO mice treated with CCl_4_ administration. Each column and error bar represents the mean and SEM, respectively, of triplicate samples. * indicates statistically significant difference at P<0.05.

As previously reported, F4/80+CD11b+ cells produced TNF-α [26]. The mRNA expression of TNF-α in hepatic tissue was significantly higher in IDO-KO mice than WT mice 24 h after single CCl_4_ administration (Fig 3). Moreover, we investigated the frequency and cell number of F4/80+CD11b+ cells in the liver under the CCl_4_-induced hepatic injury of WT or IDO-KO mice by flow cytometer. The frequency and cell number of F4/80+CD11b+ cells were significantly increased in IDO-KO mice compared to WT mice 24 hours after CCl_4_ administration (Fig 4A–4C). Next, we also examined the phenotypes of the other immune cells after CCl_4_ administration (S3 Fig). There was no difference between CCl_4_-treated WT mice and CCl_4_-treated IDO-KO mice in the frequency of CD4+, CD8+, natural killer (NK), and NKT cells in the liver. The number of these cells in IDO-KO increased compared to that in WT mice after the administration with CCl_4_.

### An increased number of α-SMA-positive HSCs in IDO-KO mice observed compared to that in WT mice

**Fig 5:**
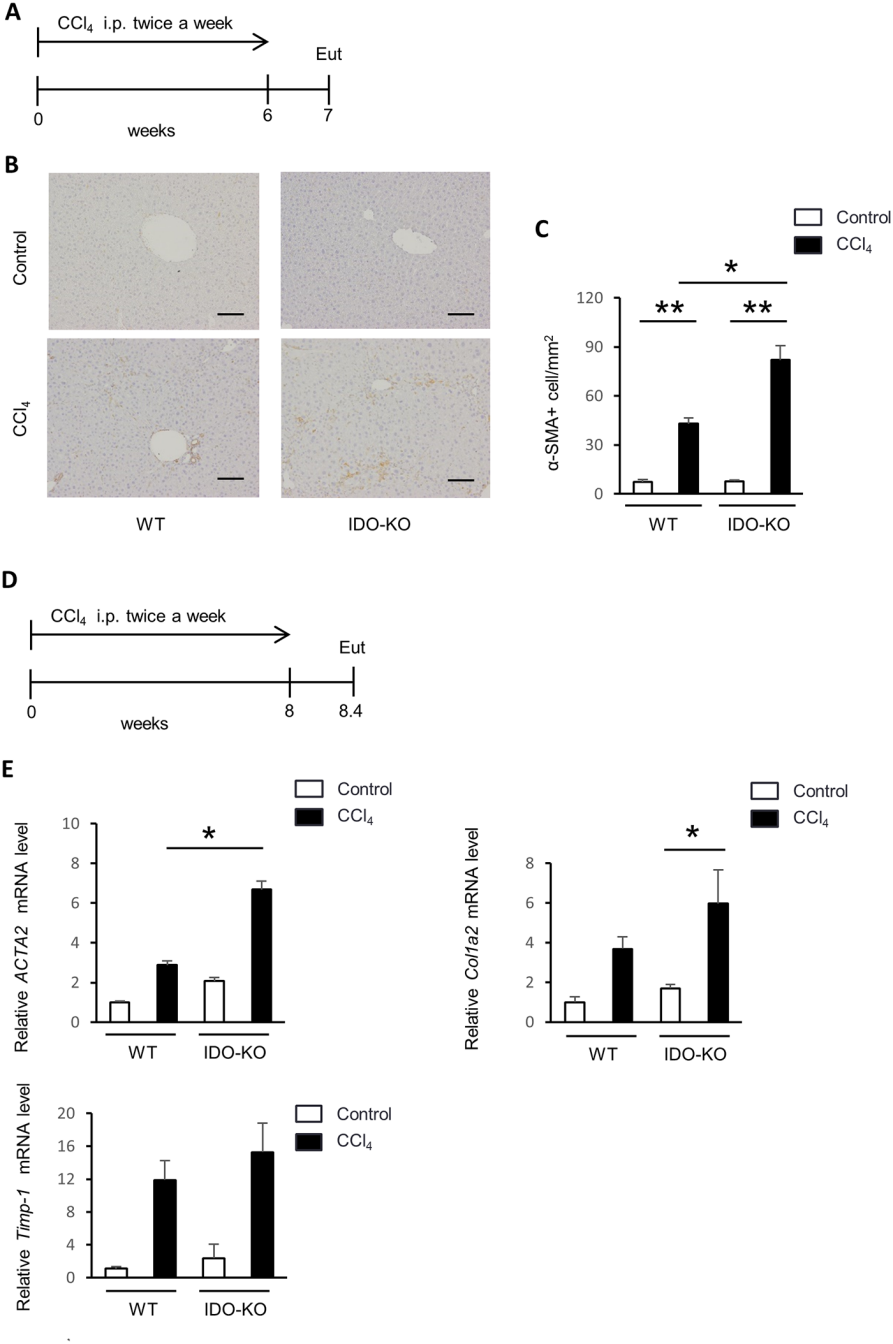
The number of α-SMA-positive HSCs was greater in IDO-KO mice compared to WT mice. WT mice (n = 4) and IDO-KO mice (n = 5) were treated with CCl_4_ (1 mL/kg, 10% diluted in olive oil) twice a week for 6 weeks. All mice were sacrificed 7 days after the last administration of CCl_4_. The control group (WT mice: n = 5, IDO-KO mice: n = 5) was injected with olive oil alone. (A) The diagram showing experimental design of Figs 5B and 5C. (B) Immunohistochemical staining of α-SMA in the liver tissue sections from the experimental mice, ×100 original magnification, Scale bar: 100 μm. (C) The number of α-SMA-positive cells per field was counted in three fields from each liver tissue. (D) The diagram showing experimental design of Fig 5E. (E) ACTA2, Col1a2, and Timp-1 mRNA expression in HSC. WT mice and IDO-KO mice administered with vehicle or CCl_4_ twice a week for 8 weeks. All mice were sacrificed at 3 days after the administration of CCl_4_ or olive oil alone. HSCs were isolated from the mice liver. The relative expression level of ACTA2, Col1a2, and Timp-1 mRNA in HSCs was measured using quantitative real time RT-PCR. The results were normalized to the expression of 18S rRNA. Each column point and error bar represents the mean and SEM, respectively, of data from triplicate samples. * indicates statistically significant differences at *P<0.05.

During the process of liver fibrosis, activated HSCs undergo increased collagen production. Because activated HSCs express α-SMA, we performed immunohistochemical staining of α-SMA in liver sections from the WT and IDO-KO mice treated with CCl_4_ repeated injection for 6 weeks. The number of α-SMA positive cells per field of the liver section was significantly greater in the IDO-KO mice compared to that in WT mice (Fig 5B and 5C).

### ACTA2, Col1a2, and Timp-1 mRNA expression in HSCs of IDO-KO mice is increased following CCl_4_ treatment compared with that of WT mice

As previously described, HSCs are activated by various pro-inflammatory cytokines [27]. Subsequently, activated HSCs express ACTA2, Col1a2, and Timp-1, and product extracellular matrix. Therefore, we assessed the ACTA2, Col1a2, and Timp-1 expression in HSCs. WT mice and IDO-KO mice were treated with CCl_4_ twice a week for 8 weeks. All mice were sacrificed 3 days after the last administration of CCl_4_. HSCs were isolated from the liver using density-gradient centrifugation. Next, we determined the mRNA expression of ACTA2, Col1a2, and Timp-1 in HSCs using quantitative real time RT-PCR. ACTA2 and Col1a2 mRNA expression was greater in IDO-KO mice compared to that in WT mice after treatment with CCl_4_ (Fig 5E).

### The administration of l-tryptophan aggravated CCl_4_-induced liver fibrosis in WT mice

**Fig 6:**
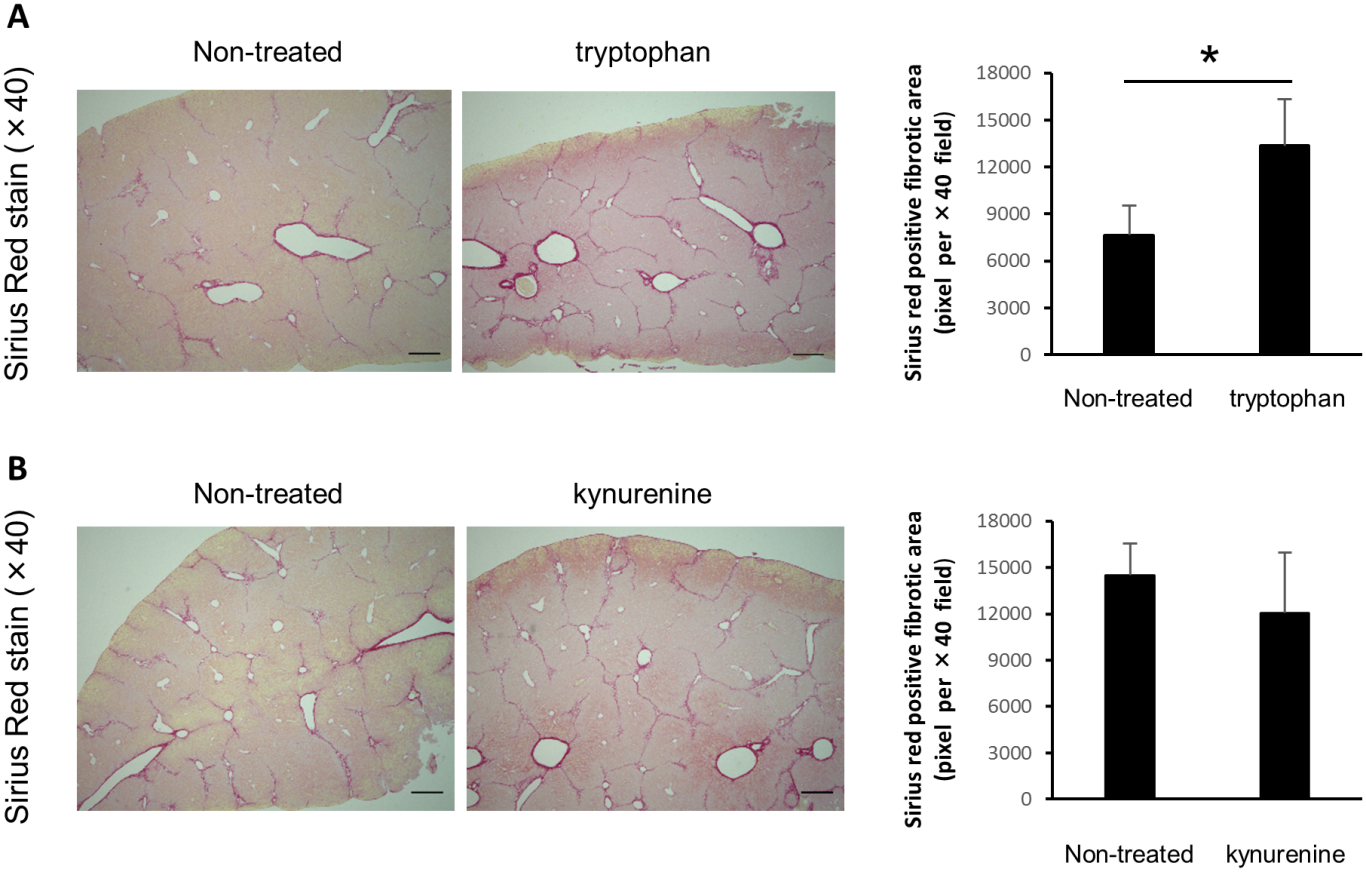
The effect of the administration with l-tryptophan and kynurenine on the development of liver fibrosis. WT mice (n = 8) were treated with CCl_4_ (1 mL/kg, 10% diluted in olive. oil) or CCl_4_ and l-tryptophan (1 mg / mouse) twice a week for 6 weeks. (B) IDO-KO mice (n = 8) were treated with CCl_4_ (1 mL/kg, 10% diluted in olive oil) or CCl_4_ and l-kynurenine (1 mg / mouse) twice a week for 6 weeks. Evaluation of fibrosis was quantified based on the Sirius red-positive fibrotic area in five random fields on the liver tissue sections for each group using ImageJ software. Each column and error bar represents the mean and SEM, respectively, of results for triplicate samples. * indicate statistically significant differences, at P<0.05.

Next, we examined the role of l-tryptophan and l-kynurenine in the development of CCl_4_-induced liver fibrosis. The administration of CCl_4_ induced the activation of IDO and decreased l-tryptophan in WT mice. On the other hand, we predict that l-tryptophan metabolites such as l-kynurenine in CCl_4_-treated IDO-KO mice are decreased compared to that in CCl_4_-treated WT mice. Therefore, to investigate the role of l-tryptophan and l-kynurenine in the development of liver fibrosis, we repeatedly administered l-tryptophan into CCl_4_-treated WT mice and l-kynurenine in CCl_4_-treated IDO-KO mice. And we stained the liver sections by Sirius red. The administration of l-kynurenine did not affect the Sirius red-positive areas in CCl_4_-treated IDO-KO mice. On the other hand, the administration of l-tryptophan significantly increased the Sirius red-positive areas in CCl_4_-treated WT mice (Fig 6).

## Discussion

In the present study, we found that hepatic fibrosis in IDO-KO mice was exacerbated by repeated administration of CCl_4_ compared to that in WT mice. CCl_4_ treatment induces various pro-inflammatory cytokines in the liver. TNF-α mRNA expression in IDO-KO mice significantly increased after single administration with CCl_4_ compared with that in WT mice. The cell number of macrophages (F4/80+CD11b+ cells), which produce TNF-α, were significantly increased in IDO-KO mice compared to WT mice after CCl_4_ administration. Moreover, HSCs in IDO-KO mice were more activated and produced more ACTA2 and Col1a2 after repeated administration with CCl_4_. Thus, the deficiency of IDO expression enhanced the development of the liver fibrosis via the activation of HSCs.

The mechanism for CCl_4_-induced liver injury and fibrosis has been studied extensively. Kupffer cells recognize dying hepatocytes induced by CCl_4_ injection, and subsequently, produce chemical mediators, such as prostaglandins, leukotrienes, platelet-activating factors, and pro-inflammatory cytokines, including TNF-α and IL-1β [28]. These signaling molecules induce the influx of leukocytes, including macrophages, neutrophils, and T cells into the necrotic area. These inflammatory cells produce cytokines that cause the proliferation of remaining viable hepatocytes as well as the transformation of HSCs into myofibroblasts to stimulate the repair process. Activated HSCs produce ECM, resulting in the liver fibrosis. Thus, the inflammatory response is a key component in liver fibrosis induced by the repeated administration of CCl_4_. In general, IDO is also related to inflammatory response because IDO expression is markedly increased by pro-inflammatory cytokines. A previous study demonstrated that the administration of CCl_4_ markedly induced IDO enzyme activity in the liver [14]. Similarly, IDO1 mRNA expression in the liver was increased in WT mice after CCl_4_ administration in the present study (Fig 1A). Moreover, CD11b-/CD11c+ cells (dendritic cells) mainly expressed IDO1 mRNA after CCl_4_ administration (Fig 1B). l-tryptophan levels in the liver tissue from WT mice were significantly reduced after CCl_4_ administration (Fig 1C). Therefore, CCl_4_ administration increased IDO expression and enhanced IDO activity in WT mice.

Recent study demonstrated that the inhibition of IDO activity by oral 1-MT administration could aggravate CCl_4_-induced liver injuries [14]. In the present study, the liver injury in IDO-KO mice treated with CCl_4_ was exacerbated compared with that in WT mice (Fig 1D–1F). CCl_4_ is metabolized primarily by the cytochrome P450 of the hepatic oxidase system to trichloromethyl radicals [29]. The trichlorometyl radicals initiate the peroxidation of polyunsaturated fatty acids in the endoplasmic reticulum and mitochondria, and destroy the biomembrane structure [30]. Cytochrome P450 have high specificity of substrate and especially cytochrome P450 2E1 (CYP2E1) metabolize CCl_4_ [31]. CYP2E1 level in the liver among WT mice and IDO-KO mice was not significantly difference (S1 Fig). Therefore, the difference of liver injury level between WT mice and IDO-KO mice after the administration with CCl_4_ might be not due to the difference of cytochrome level.

Our findings also indicated that IDO deficiency enhanced the expression of pro-inflammatory cytokines after the administration of CCl_4_ (Fig 3). Previous reports demonstrated that IDO suppressed inflammation in some experimental models [4,32,33]. Moreover, mRNA expression of PDGF-β in hepatic tissue was also significant higher in IDO-KO mice than WT mice 6 days after administration of CCl_4_. Previous report demonstrated that IDO is induced after inflammatory stimulation and suppress the immune response in the host [9]. In the inflammatory state induced by CCl_4_ injection, the expression of inflammatory cytokines increased and promptly restores to normal levels. The enhancement of cytokines expression during inflammatory states was often prolonged in IDO-KO mice [4]. In the present study, severe liver damage induced by CCl_4_ injection enhanced the inflammation-related gene expression in early time point. In IDO-KO mice, the gene expression may increase in late time point (6 days after CCl_4_ injection) because the IDO deficiency cannot induce enough immune suppression in mice. Thus, IDO deficiency enhanced the expression of pro-inflammatory cytokines and fibrogenic factors.

The increase of IDO expression has been shown to induce the decrease of tryptophan and the increase of tryptophan metabolites including kynurenine in the local microenvironment [34]. The activation of lymphocytes, specifically T cells and NK cells, requires sufficient tryptophan [10]. According to previous report, CCl_4_-induced lymphocyte infiltration to the liver was ameliorated by pre-treating with berberine and improve liver damage [35]. In present study, the number of hepatic MNCs of IDO-KO mice (Ave. 4.4×10^6^) was significantly increased compared that of WT mice (Ave. 3.1×10^6^) after 24 hours after CCl_4_ administration (data not shown). The number of hepatic MNCs of non-treat mice (WT and IDO-KO) was average 2.0×10^6^. Thus, we speculate that the deficiency of IDO increase the number of migrating lymphocyte to the liver, and aggravate liver damage. Therefore, the decrease of tryptophan in the local microenvironment suppresses the inflammatory response via the inhibition of lymphocyte activation. On the other hand, kynurenine has the ability to suppress lymphocyte activation and proliferation. The increase of kynurenine leads to the suppression of the inflammation induced by activated lymphocytes [36]. Thus, the induction of IDO suppresses the inflammatory response, and the inhibition of IDO activity aggregates the inflammation induced by activated lymphocytes. In the present study, as shown in Fig 6, the addition of l-tryptophan aggravated the liver fibrosis in CCl_4_-treated WT mice. On the other hand, the administration with l-kynurenine did not affect the development of liver fibrosis in IDO-KO mice. These results indicated that IDO deficiency induced the increase of tryptophan level in the liver, and the increase of tryptophan level might induce liver inflammation via the activation of lymphocytes and aggravate the liver fibrosis.

It is well known that inflammation is fundamentally involved in the progression of liver fibrosis in chronic liver diseases. Liver fibrosis is induced in chronic viral hepatitis, alcohol-induced hepatitis, non-alcoholic steatohepatitis (NASH) and autoimmune hepatitis. Previous studies demonstrated that the suppression of liver inflammation reduced the progression of liver fibrosis [37]. Whereas various pro-inflammatory cytokines are increased during liver injury, TNF-α has particularly been shown to be involved in these liver diseases [19]. Pro-inflammatory cytokines can induce fibrogenic factors including TGF-β [38] and Col1a2 [39]. In the present study, expression TNF-α was increased in IDO-KO mice after injection of CCl_4_ (Fig 3). In addition, the frequency and cell number of F4/80+CD11b+ cells were significantly increased in IDO-KO mice after CCl_4_ administration (Fig 4A–4C). Also, IDO1 mRNA expression was induced in CD11b- cells and CD11c+ cells by CCl_4_ administration (Fig 1B). Therefore, it indicated that CD11b- cells and CD11c+ cells expressed IDO1 by CCl_4_ administration. IDO may suppressed the inflammatory response which induced by TNF-α.

Also, the number of activated HSCs was increased and the expression of ACTA2 and Col1a2 mRNA in HSCs were up-regulated in IDO-KO mice (Fig 5B, 5C and 5E). Thus, the IDO deficiency resulted in increased liver fibrosis induced by repeated administration of CCl_4_ (Fig 2B–2D). The HSCs activation was induced by the repeated administration of CCl_4_ (Fig 5B and 5C). ACTA2 and Col1a2 expression in HSCs was enhanced following CCl_4_ injection in the IDO-KO mice (Fig 5E). Such repetitive stimulation with pro-inflammatory cytokine expression may lead to the induction of activated HSCs and the progression of liver fibrosis.

A recent study demonstrated that the induction of liver fibrosis and hepatocellular carcinoma is inhibited by the attenuation of liver inflammation during chronic viral hepatitis [40]. Previous study indicated that the IDO deficiency enhanced the liver injury in autoimmune hepatitis model (4). Our findings also demonstrated that the IDO deficiency enhanced the inflammation in the liver and aggravated liver fibrosis in CCl_4_-induced liver injury model. Taken together, enhanced IDO expression in inflammatory state may delay the progression of liver fibrosis and possibly the development of hepatocellular carcinoma.

## Supporting Information

S1 Fig mRNA expression of cytochrome in the liver tissue of WT and IDO-KO mice. The relative expression levels of CYP1a2 and CYP2e1 mRNA in the liver were measured using quantitative RT-PCR.
(TIF)

S2 Fig The effect of IDO1 inhibitor, 1-MT, in CCl4-induced hepatic fibrosis. Control group and 1-MT group (4 mg/ml dissolved alkaline water) were treated with CCl4. (A) Representative photomicrographs of experimental mice liver sections of Sirius red staining. Scale bars: 250 μm. (B) Evaluation of fibrosis was quantified based on the sirius red-positive fibrotic area in five random fields on the liver tissue sections for each group using ImageJ software.
(TIF)

S3 Fig The frequency and cell number of CD4+, CD8+, NK, and NKT cells after single administration of CCl4. (A) The frequency of CD4+, CD8+, NK (DX5+), and NKT (CD3+/DX5+) cells in the liver of WT and IDO-KO mice treated with CCl4 administration. (C) The cell number of CD4+, CD8+, NK, and NKT cells in the liver of WT and IDO-KO mice treated with CCl4 administration.
(TIF)

## Abbreviations

ECM: extracellular matrix
HSC: hepatic stellate cell
IDO: indoleamine 2,3-dioxygenase
DCs: dendritic cells
TNF: tumor necrosis factor
IL: interleukin
Tregs: regulatory T cells
CCl_4_: carbon tetrachloride
1-MT: 1-methyl-_DL_-tryptophan
WT: wild-type
IDO-KO: IDO-knockout
α-SMA: alpha-smooth muscle actin
ALT: plasma alanine aminotransferase
PBS: phosphate-buffered saline
BSA: bovine serum albumin
ACTA2: alpha smooth muscle actin2
Col1a2: collagen Type I Alpha2
MNC: intrahepatic mononuclear cell
NK: natural killer
NASH: non-alcoholic steatohepatitis
