# Supplementary figures and images for: The Deficiency of Indoleamine 2,3-Dioxygenase Aggravates the CCl4-Induced Liver Fibrosis in Mice
Source: PLoS One. 2016 Sep 6;11(9):e0162183. doi: 10.1371/journal.pone.0162183 (PMC5012673; doi:10.1371/journal.pone.0162183)

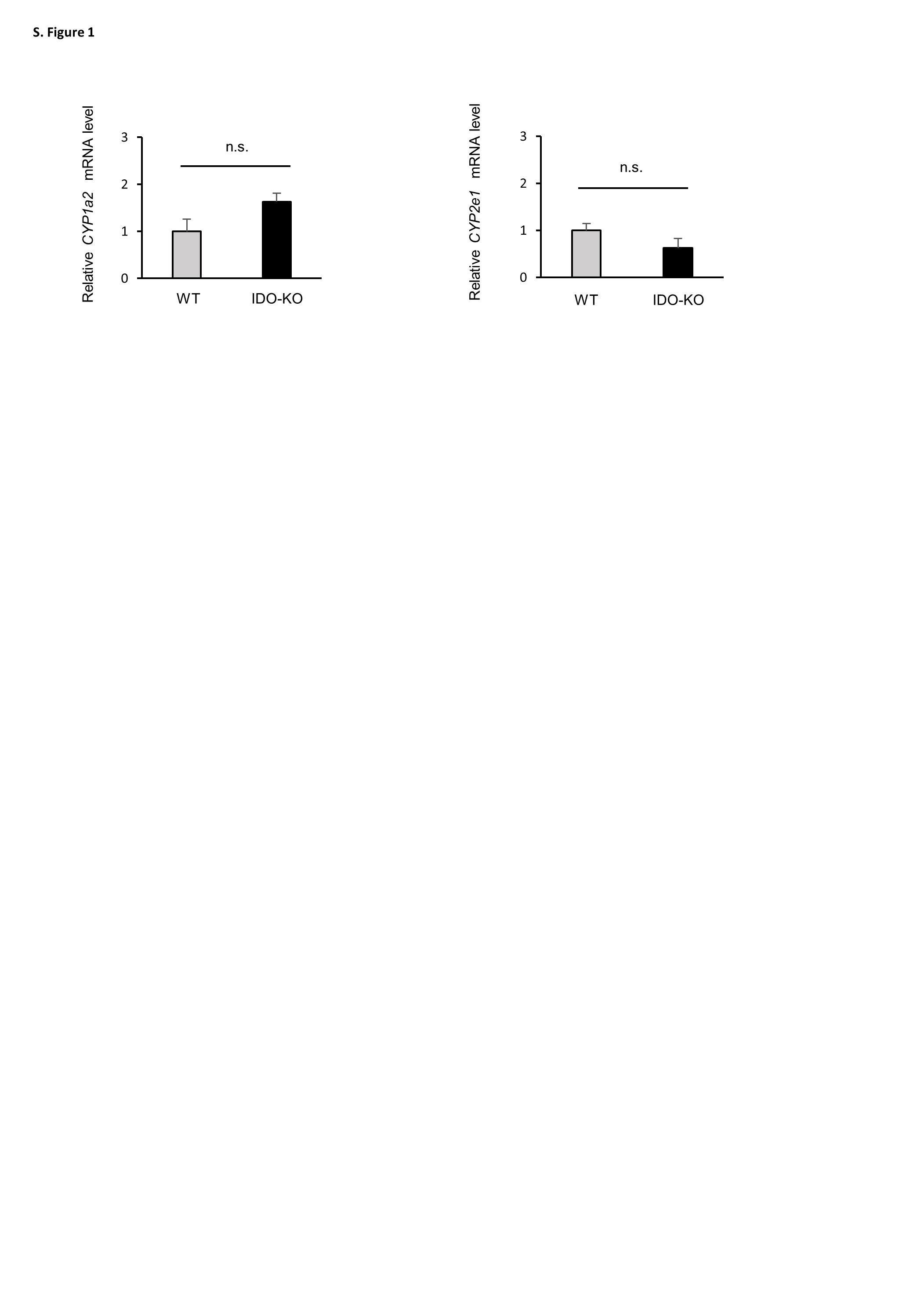

Supplement: S1 Fig — The relative expression levels of CYP1a2 and CYP2e1 mRNA in the liver were measured using quantitative RT-PCR. (TIF) [file pone.0162183.s001.TIF]

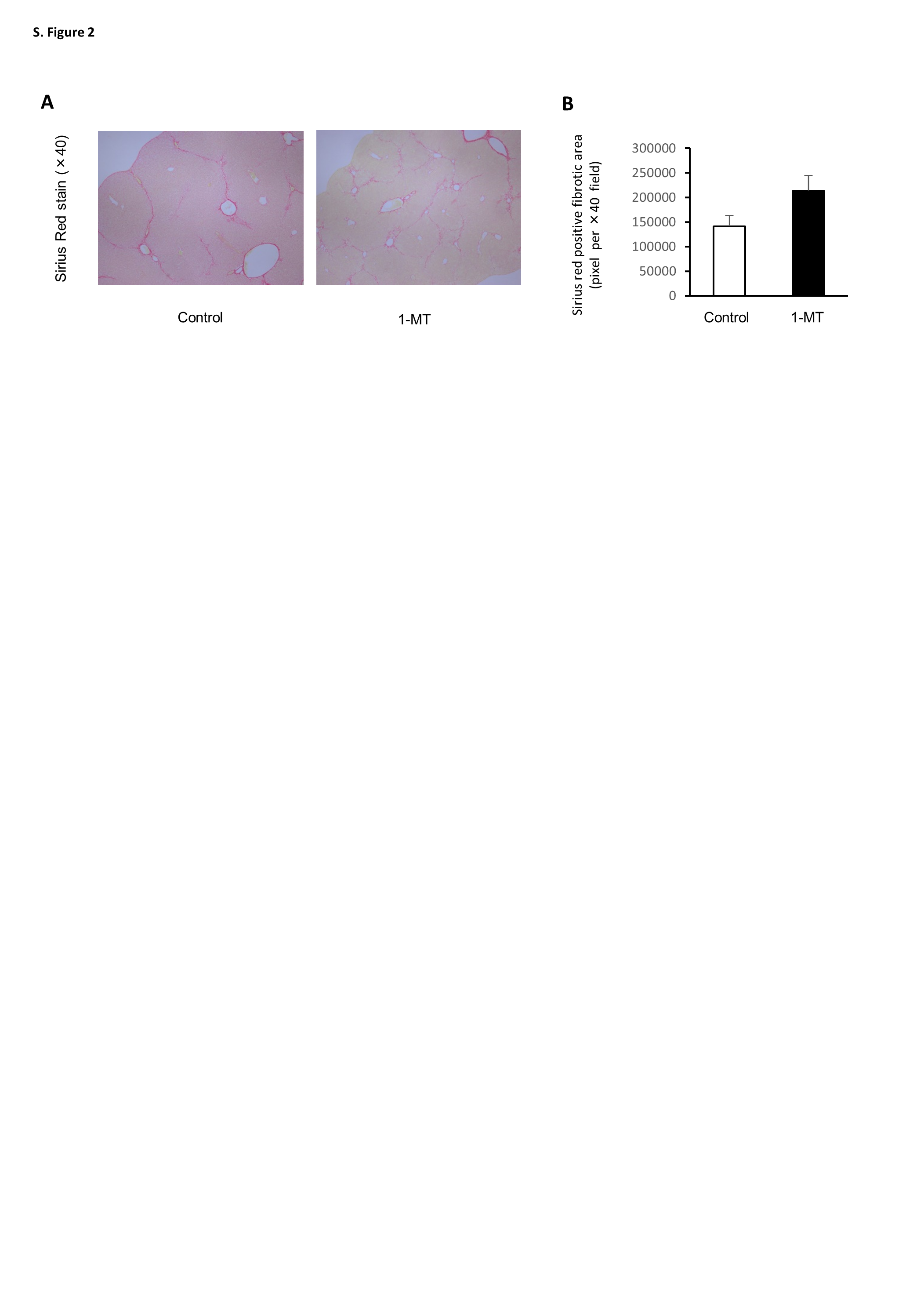

Supplement: S2 Fig — Control group and 1-MT group (4 mg/ml dissolved alkaline water) were treated with CCl4. (A) Representative photomicrographs of experimental mice liver sections of Sirius red staining. Scale bars: 250 μm. (B) Evaluation of fibrosis was quantified based on the sirius red-positive fibrotic area in five random fields on the liver tissue sections for each group using ImageJ software. (TIF) [file pone.0162183.s002.TIF]

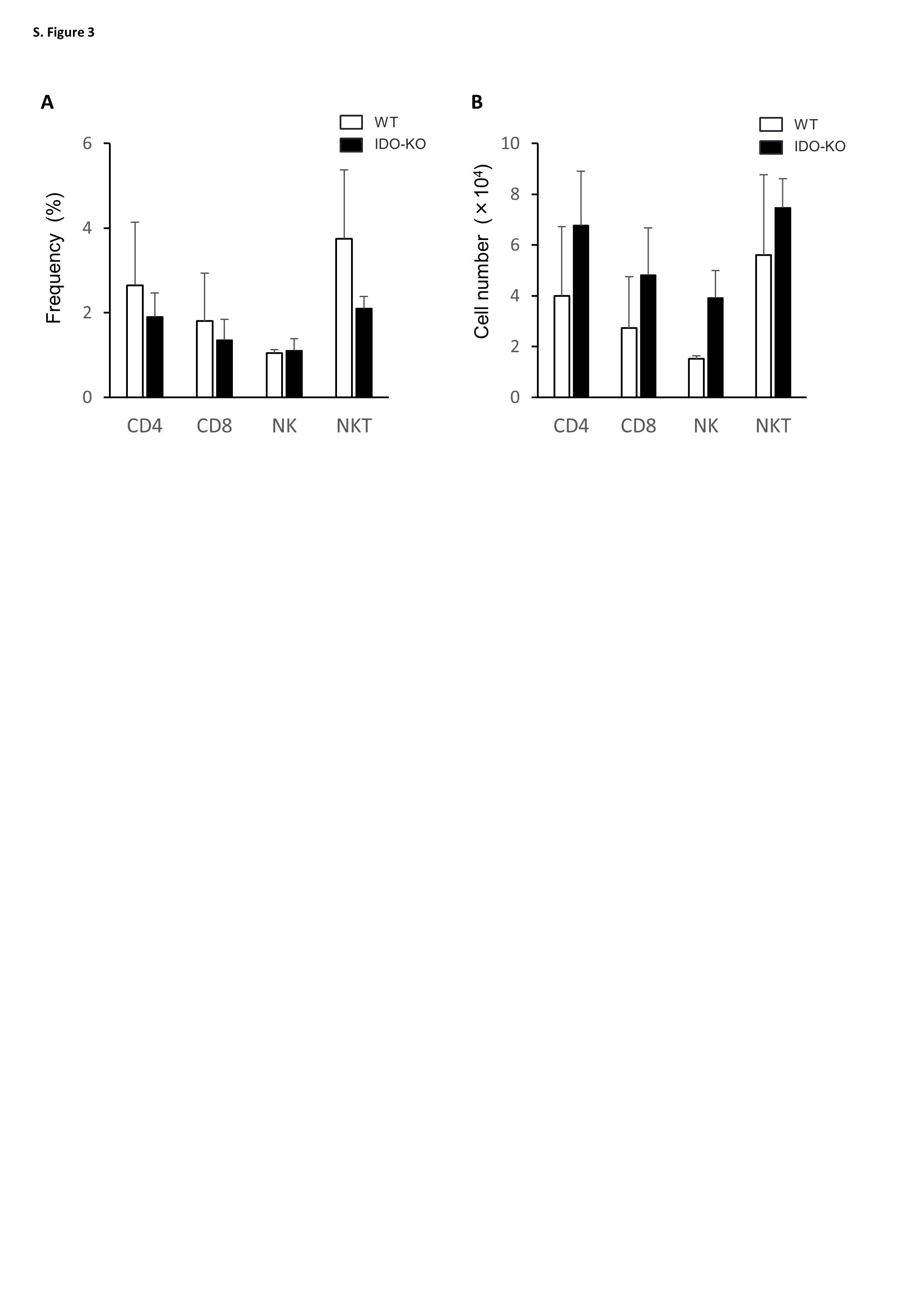

Supplement: S3 Fig — (A) The frequency of CD4+, CD8+, NK (DX5+), and NKT (CD3+/DX5+) cells in the liver of WT and IDO-KO mice treated with CCl4 administration. (C) The cell number of CD4+, CD8+, NK, and NKT cells in the liver of WT and IDO-KO mice treated with CCl4 administration. (TIF) [file pone.0162183.s003.TIF]
